# A Playroom Internal Waiting Area Improves Productivity in the Pediatric Emergency Department

**DOI:** 10.5811/westjem.2019.10.43413

**Published:** 2020-02-21

**Authors:** Paul Walsh, Jennifer Denno

**Affiliations:** Sutter Medical Center, Department of Pediatric Emergency Medicine, Sacramento, California

## Abstract

**Introduction:**

Pediatric emergency department (PED) volume is often constrained by the number of available treatment rooms. In many PEDs patients occupy treatment rooms while awaiting test results or imaging, thereby delaying care for patients who arrive after them.

**Methods:**

We opened a PED where selected patients were moved to a playroom when they did not actively require a treatment room. The treatment room was then available for the next patient. We measured the effect of using the playroom on time from arrival to rooming and length of stay (LOS) using proportional hazards regression and the odds of being roomed within 30 minutes of arrival using logistic regression. We adjusted for the number of the previous eight patients who were “playroom eligible”; age; triage category; provider; the number of patients who arrived within the preceding hour; prior census; and testing ordered in the preceding eight patients.

**Results:**

We analyzed 43,634 patient encounters, of which 10,134 (23%) were playroom eligible. The adjusted hazards ratio for the next patient being roomed was 1.14 (95% confidence interval [CI], 1.10–1.18) per prior playroom eligible patient. The adjusted odds ratio of the next patient being roomed within 30 minutes was 1.46 (95% CI, 1.33–1.56) per prior playroom eligible patient. The playroom typically decreased median rooming time by four to 42 minutes and LOS by two to 40 minutes depending on patient volumes and acuity. The benefit of the playroom was maximal at busier times.

**Conclusion:**

Implementing a playroom in the PED for selected patients generally decreased time to rooming of the next patient and LOS.

## INTRODUCTION

Rapid rooming of patients on arrival facilitates clinical decision-making and disposition, thereby increasing the number of patients a pediatric emergency department (PED) can see per hour; rapid rooming also improves the perception of care and timeliness. Parents value being seen quickly on arrival in the PED and, particularly in non-monopoly markets, this is important. Rapid rooming, however, requires empty treatment rooms, and these are typically limited by physical or staffing constraints.^1^–^4^ Efficiently using the available staffed spaces becomes paramount. Here we describe how we measured the effect of a PED playroom on time to rooming of patients and total length of stay (LOS).

We have observed that in many PEDs most of the time in treatment rooms is spent waiting, rather than being treated or evaluated. Such waiting is typically for patient registration, imaging to be performed, test results to return, and antipyretics to take effect. While waiting, the treatment room itself adds no value to the child’s stay. Worse, treatment rooms are designed for clinical care, which is inherently child unfriendly. Frequently, parents spend a good deal of time restraining their child’s natural curiosity, adding to the stress of the encounter. The opportunity cost to keeping patients in treatment rooms for the duration of their ED stay is that it prevents other children from being seen. Despite this, there is a widespread culture in many American PEDs of keeping children in treatment rooms for the duration of their ED visit.

## METHODS

We created a flow system moving children who were not receiving active interventions from their treatment room to a playroom. This space is child friendly and, as with inpatient playrooms, examinations and procedures are prohibited in this “safe space.” Children in the playroom are supervised by their parents, not nursing staff. This frees up nursing staff and treatment rooms to allow the next patient to be evaluated. We are unaware of any prior attempts to implement such a playroom model in pediatric emergency medicine.

Randomized controlled trials of interventions such as ours are impractical; the numbers of PEDs being opened is simply too small and the prospect of obtaining consent from hospital administrators to allow their PED to be randomized to a potentially less-efficient flow model is remote. Before and after studies are difficult because the concept is unproven and secular effects are inevitable. Implementing and comparing alternate patient-flow systems on alternate days presents logistical challenges and costs that few healthcare systems would contemplate. Consequently, we tried to demonstrate the effect of a PED playroom on patient flow by comparing patient flow characteristics at times when the playroom model could be of benefit compared with times when we knew by the limits of our design that a playroom model could not help. We would then attribute the difference in performance primarily to the playroom.

This study was exempt from institutional review board review.

### Setting

This was a community PED seeing 21,000 patients annually at the time of the study with mixed pediatric/general emergency physicians and advanced practice provider (APP) staffing model. The PED has 11 exam rooms with a guaranteed minimum staffing for eight beds and sees patients up to 21 years of age.

### Study Definitions

Time zero was set as the time the patient was entered into the computer system. This was performed by a nurse in the arrival lobby for patients who were brought in by their parents and by the nursing team leader if a patient was brought in by ambulance. We measured the time interval from arrival (time zero as defined above) to either (1) being roomed by a nurse or (2) roomed and evaluated by a physician or an APP, whichever was shorter. This analysis method captured cases where the medical exam was initiated before or during the nursing triage process. We defined LOS as time from arrival to time the patient left the ED.

For analysis purposes we derived playroom eligibility from recorded electronic health record (EHR) variables and the fact of being placed there. This assumes that children who were not placed in the playroom were ineligible to be placed there for subjective reasons (eg, medically or behaviorally unsafe, rather than staff not moving them).

**Population Health Research Capsule**

What do we already know about this issue?

Children occupying treatment rooms in the pediatric emergency department while awaiting test results or to defervesce delays the evaluation of subsequent children.

What was the research question?

What would be the effect of moving these children from treatment rooms to a shared playroom?

What was the major finding of the study?

A playroom internal waiting area improved throughput times overall except during very quiet times.

How does this improve population health?

In cultures where parents expect to occupy a treatment room for the duration of their child’s stay, incorporating a playroom improves patient throughput times.

### Outcomes

Our primary outcome was the effect of a playroom on rooming times measured by the hazard ratio (HR). We measured the effect of the playroom on the odds of a patient being roomed within 30 minutes of arrival. Our secondary outcome was the effect of playroom use on overall PED LOS. For this we measured the interval from rooming to discharge and added it to the interval from arrival to rooming.

### Intervention

The intervention was a PED playroom where patients could await the next task in care. Patients were classified as eligible to be placed in the playroom (“playroom eligible”) if they met all the following criteria: required only imaging, urine testing, or venipuncture without intravenous placement; older than eight weeks; not immunocompromised; and not suspected to be medically or behaviorally dangerous to other children. (For example, a suspected case of measles or a child prone to violent outbursts could not be sent to the playroom.) Children not meeting these criteria were “not playroom eligible.” Children who were “not playroom eligible” had, except for trips to the radiology suite, to be kept in their treatment rooms for the entire duration of their ED stay. Staff, not parents, determined playroom eligibility.

The PED patient-flow model expected immediate rooming and in-room triage by the nurse assigned to an exam room unless all exam rooms were occupied. Any team member could room a patient; physician evaluation could occur before, during, or after nursing triage. Nursing triage as in most EDs performs a variety of functions in addition to determining treatment priority. Prior to implementation we trained a core group of nurses who staff the PED and provided immediate feedback when the model was not being implemented. We used nurse staff meetings and weekly electronic newsletters to reinforce use of this model during the initial year.

### Analysis

We performed a retrospective analysis using data from all PED visits extracted from the EHR from August 8, 2015, to August 8, 2017. We performed regression parameterized as a proportional hazards model with the Gompertz distribution using Stata 14.2 statistical software (Statacorp LLP, College Station, TX).^5^ We adjusted the regression for patients’ ages and triage category; individual physician or APP; the number of patients who arrived within the preceding hour; whether any laboratory testing was performed; how many of the preceding eight patients required lab testing. We used the previous eight patients due to the PED’s minimum staffing for eight beds. We tested for interactions between variables and retained those that were important.

We included a cluster term for patient to adjust for repeat attendance. We also included a variable for the initial 11-month period when the PED functioned as a discrete unit embedded within an adult ED with limited physical barriers. After this period the PED was relocated within the existing space by bed re-designation and physically separated from the adult unit with three sets (rather than the previous single set) of double doors, and provided with its own ambulance entrance. This change added several minutes walking time for parents from arrival (time zero) to their treatment room.

We graphed the proportional hazards regression to show the effects of the playroom on median time to rooming under differing patient acuity and volume scenarios. These graphs allow the reader to compare scenarios when there were no playroom-eligible patients and when there were more rooms than patients available (ie, the playroom could not affect patient throughput) and with a range of other scenarios when a playroom could improve patient throughput. We used logistic regression, with the same independent variables as the proportional hazards model, to estimate the odds of a patient being roomed within 30 minutes of arrival. The differences observed between these scenarios reflects the effect of the playroom on patient throughput.

For our secondary outcome, we created a proportional hazards regression model of the interval from being roomed to leaving the ED. This prevented incorporation of the direct effects of the playroom noted in the first regression contaminating the second regression. Variables that lead to faster rooming (e.g., higher acuity) may also lead to longer time to discharge. We included the interval from arrival to rooming as an independent variable to see whether there were any indirect effects of changing the time to being roomed on the subsequent duration of the visit. We also included age, triage category, and blood, urine or radiology testing, as independent variables.

We tested for interactions between variables and retained those that were important, including three-way interactions between the number of patients arriving in the PED during that hour, on that day, and the number of the preceding eight patients who were playroom eligible. We again included a cluster term for patient to adjust for repeat attendance. This model better reflected reality than simpler models and allowed for the possibility that the playroom intervention could variably improve or worsen rooming times depending on circumstances. Because of this variable effect, we graphed the effect of the playroom under different scenarios using the marginsplot function in Stata. We estimated the effect on our secondary outcome indirectly using the median time taken to room patients from the second regression (indirect effect) and adding the resulting median time to the time to be roomed (direct effect). We manually graphed our secondary outcome under a selected number of scenarios.

## RESULTS

We had 43,634 patient encounters of whom 10,134 (23%) were playroom eligible and 2,260 (5%) were admitted. Table 1 summarizes the demographic characteristics. The adjusted hazards ratio (HR) of rooming from arrival was HR 1.14 (95% confidence interval [CI], 1.10–1.18) per previously arriving playroom eligible patient. There were significant interactions between the HR for initial rooming, the total number of patients seen that day (started at midnight) up to the arrival of the current patient, and the number of patients who arrived within an hour of the patient arriving. The odds ratio (OR) of a patient being roomed within 30 minutes of arrival was OR 1.46 (95% CI, 1.33–1.56) for each previously arriving playroom-eligible patient.

The impact of the playroom on PED LOS varied depending on daily census and recent arrivals. For example, during a quiet period (10 patients seen before the current patient, of whom only two presented within an hour of the current patient), the decrease in PED rooming time, due to four vs zero playroom-eligible patients, was four minutes (10 vs 14 minutes) and overall improvement in LOS was two minutes (96 vs 98 minutes). In sharp contrast, when the department was busy (90 patients seen before the current patient, 12 of these presented within an hour of the current patient), the decrease in PED rooming time, due to four vs zero playroom-eligible patients, was 42 minutes (68 vs 110 minutes), and the overall improvement in PED LOS was 40 (168 vs 208) minutes.

Table 2 shows the effects of each variable and their interactions. Higher acuity in the current patient, lower acuity in the preceding eight patients, and fewer investigations in the preceding eight patients were associated with shorter median rooming times. Conversely, lower acuity in the current patient being treated, higher overall census, and more patients arriving within an hour of the current patient, were all associated with longer median rooming times.

**Figure 1:**
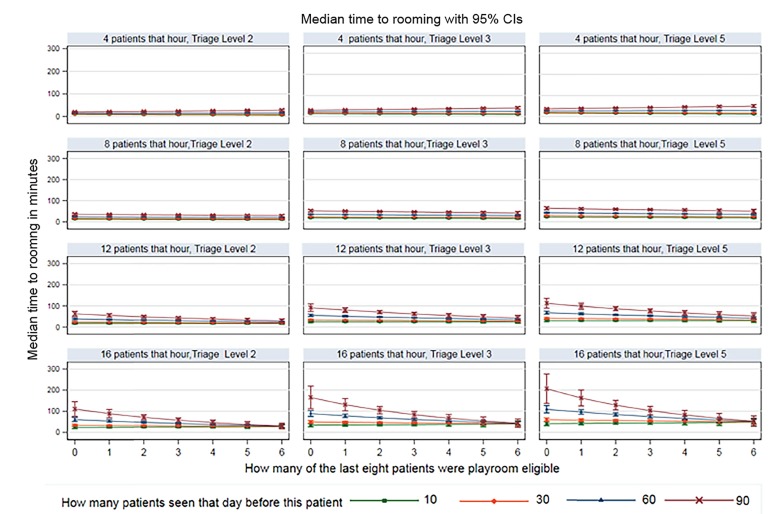
Plots showing the effect of increased numbers of playroom eligible children. *CI*, confidence interval.

**Figure 2:**
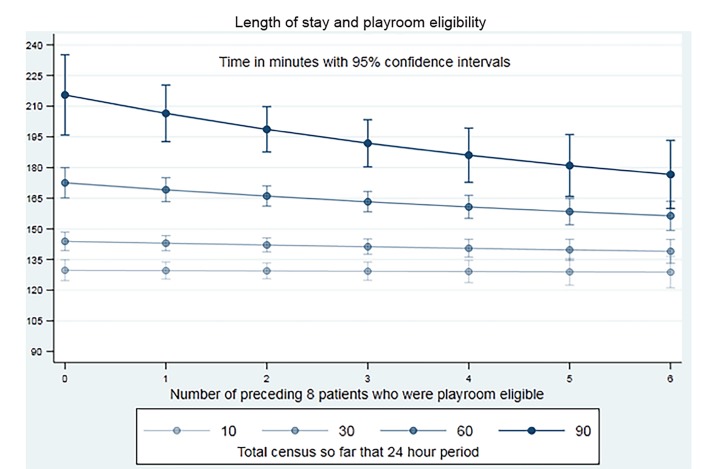
Plots showing the effect of the playroom on overall length of stay.

Figure 1 shows the effects of using a playroom/internal waiting room model given various scenarios. These graphs show decreased median time to rooming as the number of playroom-eligible patients increases. As patient census increases, particularly when a large number of patients arrive in the hour preceding the arrival of the current patient, the median time to rooming increases, despite increasing numbers of playroom-eligible children. This reflects the point where the number of patients to be seen exceeds staff capacity. Figure 2 demonstrates the effect of the playroom on total LOS in a subset of scenarios presented in Figure 1.

Table 3 shows that the interval between being roomed and being discharged was most heavily influenced by the severity of illness and the extent of laboratory and radiological testing performed on the child him/herself rather than on the investigation testing ordered on other children. We found an association between shorter time to discharge after being roomed and the log(*e*) of the interval between arrival and being roomed (Table 3). This partially offsets the reduction in time to rooming on overall length of stay in the PED and the overall effect of the playroom model varies with increasing PED activity.

## DISCUSSION

The answer to the question, “Does a playroom decrease time to rooming and LOS?”, is that it depends. The playroom intervention generally decreased patient rooming and LOS times. The effect size varies with how busy the PED is; up to a point, the busier the PED the greater the benefit. When all treatment rooms are filled with non-playroom-eligible patients then the benefit of the playroom disappears. Times to rooming and ED LOS under this scenario reflect the benefit of the playroom and other patient characteristics. Our results adjust for these other characteristics to the extent that we could, but our estimates remain just that.

Conversely, when patient volumes are low, moving patients to the playroom (for example, to defervesce) and sometimes having to move them back to a treatment room for re-evaluation imposes a time cost without clear benefit to the next patient who has not yet presented. The practical implication is that during quiet times, typically 3 am to 8 am in our PED when there are open available exam rooms, patients can be allowed to sleep in an exam room without loss of productivity.

Our other findings, higher acuity in the current patient, and lower acuity and less laboratory testing in preceding patients, was associated with more rapid rooming seem self-evident but their magnitude is important. While acuity cannot be changed, implementation of evidence-informed pathways and additional physician training may decrease reliance on laboratory investigations and thereby further improve patient throughput.

While improving flow in the PED is primarily a PED priority, flow is dependent on many factors that the PED cannot easily control, such as staff and actual or functional space limitations in both the PED itself and in inpatient services.^6^,^7^ Our approach facilitates early clinical decision-making; this is particularly effective at decreasing LOS.^4^ Interventions such as those that can be implemented by the PED itself are particularly desirable.^8^,^9^ Decreasing waiting times and LOS decreases the number of patients who leave without being seen and improves patient satisfaction.^10^ Parents generally accept this approach. We have found that comparing our approach to Southwest Airlines boarding is both apt and readily accepted.

Our approach fits squarely within the overall strategy of “internal waiting rooms” and “awaiting results” areas used in general EDs. Our data provides objective supportive evidence for general ED directors who wish to implement an internal waiting room. There are unique imperatives to PED playrooms, however. First, a playroom addresses much of the challenge of child-centered care in the ED. Second, it helps decrease parental anxieties as they see their child defervesce and resume normal behavior. In some settings parents’ notions of suitability of their children’s newly-found playmates may occasionally arise, although in our experience this is rarely verbalized.

This work builds on the underlying time and space limitations thesis of Michelson et al. We effectively created more treatment space and nursing resources by removing those children who need neither from the treatment room.^7^ There are limits to what our playroom model can achieve as evidenced by a small offset in the benefit of rapid rooming on the time taken in the next phase of care. This may reflect a difference in settings. Michelson et al. describe an academic setting where doctors are plentiful; in the community setting there are far fewer medical providers delivering more care. Second, in Michelson et al space was a limiting factor 5% of the time. In our setting we anticipated a daily attendance of up to 45 patients a day, but in practice have seen 110 on busy days. This distinguishes our observed practice from Michelson et al’s computer models. The underlying principles guiding their models and our intervention are the same. Other concerns include that increasing PED efficiency results in sufficiently increased census that downstream resources (eg, lab, inpatient services) may find their workload increased.

Ideally, the playroom is a separate physical space with primary stewardship belonging to child life services (play therapy). However, the same benefits could be expected to be obtained by simply moving patients back to the waiting room without the investment in child-centeredness implied by the playroom model. Whether it would be as well accepted by parents depends on the setting. In our case the opposite occurred. Our census is now substantially higher than the 14,500 patients originally planned for when we designed a “no wait” PED. Consequently, some patients now do have to wait to be roomed, and the playroom space is often shared with some patients who have just arrived.

Using the playroom requires PED staff to empower the parents to observe their children as they defervesce, or as part of a head injury observation period, secure in the knowledge that PED staff are immediately available should they be needed. Empowering parents in this way teaches them how to manage simple fevers at home and reassures them in the face of a common tendency to overestimate how sick one’s own child is. It also reduces the overall cost of care by allowing staff to see other patients. When a strategy of parental observation in the playroom is used, such as for head injuries, staff need to recognize that interval development of symptoms may require rapidly returning a patient to a treatment room. This is to be expected and should not be interpreted as a failure of the approach.

The concept of playroom/internal waiting area is straightforward, but successful implementation required intensive prolonged effort by physician and nurse leaders with wholehearted support from hospital administrators. This process results in more patients being seen in a shift by the same number of staff. Consequently, these staff need to be supported. Although beyond the scope of the evidence presented here, we observed that additional physician training, with PED management protocols to relieve cognitive load, order sets or order preference lists that align with PED management protocols, and physician scribes are all hugely helpful when implementing this approach. Nursing staff need to be similarly given additional training and supported with respect to streamlining processes and documentation that do not add value to the patient. A key investment is a child life specialist (CLS). We initially relied on inpatient CLS staff, but as their value became clear we brought in two of our own CLS as part of our PED staff.

Future research could focus on refining playroom eligibility, measuring associated parallel flow strategies, the effects of CLS specialists, and reinventing nursing processes, the role of discharge instructions, and in identifying those processes that parents perceive as adding little value.

## LIMITATIONS

This was a single center where the model was implemented at the inception of the PED, prior to the establishment of a culture that would allow unnecessary in-room waiting by patients. It is intuitive that the time taken to room a patient could be affected by the number of patients seen that day, that hour, as well as by the acuity and laboratory testing required for the prior patients. Statistical models risk oversimplifying this reality. We have addressed this by using interacted models which, although more complex than parsimonious ones, had better fit characteristics and more faithfully reflect the observed reality. These complex models require graphical description to be readily understood. Even these models are simplifications of reality.

Proving causality is difficult; our approach of comparing time to rooming and ED LOS and when the PED playroom can work (open treatment rooms) and cannot work (all treatment rooms occupied with non-playroom eligible patients) is an estimate, which despite adjustment for other considerations, will always be influenced by patient load and complexity. Nonetheless, given the constraints inherent in this type of research our estimates have been estimated as tightly as possible.

Our results also occur in the context of parallel flow where any team member can room a patient and use an electronic tracking board to communicate that fact. This parallel flow decreases the potential for the triage process to impede overall PED productivity. This effect is approximated in other PEDs by employing multiple nurses dedicated to initial triage. Parallel rooming and a playroom/internal waiting area represent different independent processes, and the former does not alter our findings about the latter. Our work does not address other factors in PED operations and patient satisfaction such as quality of patient-staff interactions, perceptions of caring, and time spent with patients.^11^,^12^

We were limited in the variables we could use. Triage category, although used for prioritizing patients, is relatively crude. We accept that some readers may regard our secondary outcome as more important than our primary outcome. We also did not perform a chart review to determine appropriateness of the decision-making as to which children were moved to the playroom. As a group, playroom-eligible children were less sick, older, and had less laboratory testing than those who were not.

## CONCLUSION

Implementing a playroom in the PED for selected patients generally decreases time to rooming of the next patient and decreases LOS.

## Figures and Tables

**Table 1:** Demographic description comparing patients who were and were not playroom eligible (total not always 100% due to rounding).

	Not playroom eligible	Playroom eligible
N	33,500	10,134
Age by category		
Neonate	1,057 (3.2%)	0 (0.0%)
1–12 months	4,796 (14.3%)	1,364 (13.5%)
1–5 years	11,343 (33.8%)	3,079 (30.5%)
6–12 years	6,162 (18.4%)	2,063 (20.4%)
13–17 years	5,238 (15.6%)	2,009 (19.9%)
18–21 years	4,928 (14.7%)	1,595 (15.8%)
Gender		
Male	16,387 (48.9%)	5,213 (51.4%)
Triage level (Level 1 most severe)		
Level 1	30 (0.1%)	0 (0.0%)
Level 2	2,159 (6.7%)	0 (0.0%)
Level 3	10,783 (33.6%)	3,520 (35.2%)
Level 4	16,535 (51.5%)	6,323 (63.2%)
Level 5	2,632 (8.2%)	161 (1.6%)
Minutes, arrival to room, median (IQR)	16 (8, 34)	18 (8, 38)
Roomed ≤15 minutes of arrival	14,644 (46.1%)	4,411 (44%)
Roomed ≤30 minutes of arrival	22,740 (71.5%)	6,931 (69.6%)
Admitted	2,122 (6.3%)	138 (1.4%)
Number of previous 8 patients who were playroom eligible, median (IQR)	2 (1, 3)	2 (1, 3)
Number of previous 8 patients who had no testing, median (IQR)	4 (3, 6)	4 (3, 5)
Number of previous 8 patients who had no urinalysis, median (IQR)	1 (0, 1)	1 (0, 1)
Number of previous 8 patients who had blood drawn, median (IQR)	2 (1, 2)	2 (1, 3)
Number of previous 8 patients who had imaging ordered, median (IQR)	2 (1, 4)	3 (2, 4)

*IQR*, interquartile ratio.

**Table 2:** Effect of playroom and other independent variables on time to rooming.

	Time to rooming (HR)	95% CI (lower, upper)	Roomed <30 min (OR)	95% CI (lower, upper)
Current patient variables				
Age in years (per year)	0.986	(0.984, 0.987)	0.980	(0.977, 0.984)
Triage (Level 3 is referent)				
Level 1	1.795	(1.108, 2.907)	3.143	(0.646, 15.296 NS)
Level 2	1.448	(1.380, 1.520)	2.079	(1.810, 2.400)
Level 4	0.827	(0.808, 0.847)	0.614	(0.582, 0.647)
Level 5	0.826	(0.793, 0.860)	0.573	(0.520, 0.631)
Variables for preceding eight patients				
No testing ordered (per patient)	1.146	(1.071, 1.227)	1.067	(1.044, 1.090)
Mean triage level (per category higher means lower acuity)	1.310	(1.195, 1.437)	1.313	(1.213, 1.422)
Playroom eligible (per patient)	1.136	(1.095, 1.178)	1.458	(1.329, 1.560)
Number of patients seen since 00:00 that day (per 10)	0.936	(0.918, 0.954)	0.875	(0.831, 0.917)
Number of patients that hour	0.940	(0.923, 0.956)	0.911	(0.874, 0.948)
Interacted variables. Effects are shown in Figure 2				
Playroom eligible X total patients that day (per 10)	0.970	(0.961, 0.978)	0.926	(0.905, 0.948)
Playroom eligible X total patients that hour	0.989	(0.981, 0.994)	0.963	(0.945, 0.982)
Total patients that day (per 10) X total patients that hour	0.992	(0.989, 0.996)	0.975	(0.965, 0.985)
Three-way interaction of above terms	1.004	(1.002, 1.005)	1.010	(1.006, 1.015)
Seen when PED beds were nearer the main entrance	0.942	(0.922, 0.963)	0.873	(0.832, 0.917)

The first model shows the hazard ratio for time to being roomed after arrival. The second model shows the odds of being roomed within 30 minutes of arrival and although less informative may be easier to operationalize than the first. Retaining the interacted variables fits the observed data better than a parsimonious approach.

*HR*, hazards ratio; *CI*, confidence interval; *OR*, odds ratio; *min*, minutes; *NS*, not significant at p <0.05.

**Table 3:** Regression model of variables affecting time from rooming to discharge.

Variables	Hazard ratio	95% CI (lower, upper)
ln (Age in years)	1.020	(1.014, 1.025)
Triage Category 1	0.310	(0.213, 0.450)
Triage Category 2	0.173	(0.151, 0.198)
Triage Category 3	referent	
Triage Category 4	1.434	(1.396, 1.472)
Triage Category 5	1.985	(1.910, 2.063)
ln_ (Arrival to rooming)	1.065	(1.052, 1.078)
Total census (per 10 patients)	0.966	(0.960, 0.971)
Blood test	0.523	(0.507, 0.540)
Urinalysis	0.708	(0.661, 0.758)
Imaging	0.751	(0.731, 0.772)
Blood test X urinalysis	1.201	(1.083, 1.332)
Imaging X Triage Category 1	1.741	(0.443, 6.849)
Imaging X Triage Category 2	3.811	(2.987, 4.861)
Imaging X Triage Category 3	referent	
Imaging X Triage Category 4	0.809	(0.782, 0.836)
Imaging X Triage Category 5	0.588	(0.536, 0.645)

This demonstrates a small indirect offset of the benefit of the effect of faster initial rooming. However, the overall time saving in faster initial rooming more than compensates for this offset.

*ln_;* natural log of; *CI*, confidence interval.
